# Lacrimal gland adenoid cystic carcinoma: a rare case report

**DOI:** 10.3389/fopht.2026.1936845

**Published:** 2026-10-29

**Authors:** Estefania Ramírez Marquez, Maria Garcia Forestier, Maria Correa, Joseph Campbell

**Affiliations:** 1 Ophthalmology, University of Puerto Rico School of Medicine, San Juan, Puerto Rico; 2 Pathology and Laboratory Medicine, University of Puerto Rico, San Juan, Puerto Rico

**Keywords:** adenoid cystic carcinoma, case report, exenteration, lacrimal gland, malignancy

## Abstract

Lacrimal gland adenoid cystic carcinoma (LGACC) is a rare and aggressive malignancy characterized by perineural invasion, late recurrence, and high mortality. No consensus treatment guidelines exist, and management strategies range from eye-sparing excision to orbital exenteration combined with chemotherapy and radiotherapy. This report describes a 32-year-old Hispanic man with a six-year history of right orbital pressure that progressed to pain, diplopia, and lacrimation. Clinical examination revealed 20/20 visual acuity bilaterally, a palpable right upper eyelid mass, and hypoglobus with inferonasal globe displacement. Imaging identified a well-defined right lacrimal gland mass, and biopsy confirmed adenoid cystic carcinoma. Intra-arterial chemoreduction was not feasible; however, three cycles of systemic cisplatin and doxorubicin resulted in no radiographic tumor reduction. The patient subsequently underwent right orbital exenteration, followed by adjuvant radiotherapy, and remains under multidisciplinary surveillance, with no evidence of local recurrence at 34 months of follow-up. LGACC is best managed with an individualized, interdisciplinary, and multimodal approach. Given the lack of consensus guidelines and the risk of late recurrence, long-term monitoring is essential, and further research is required to establish surveillance and management strategies.

## Introduction

Lacrimal gland tumors occur in fewer than one person per million annually (1); adenoid cystic carcinoma accounts for approximately 60% of malignant epithelial lacrimal gland tumors (2, 3). This malignancy is recognized for its aggressiveness, proclivity for perineural invasion, and a high mortality rate (1–4). There are no well-defined treatment guidelines for lacrimal gland adenoid cystic carcinoma (LGACC), with the existing literature predominantly comprising isolated case reports due to the rarity of this malignancy (2, 4). Most reports refer to a multimodal approach and treatment combining surgery, radiotherapy, and chemotherapy (1).

Controversy exists regarding treatment strategies, as some authors advocate for local lesion excision to preserve the eye, and others advocate for more aggressive management involving orbital exenteration (1–3). Moreover, LGACCs follow an unpredictable clinical course that is characterized by a propensity for late recurrences and distant metastases, which further underscores the urgency of comprehending this disease (4). We hereby present the case of a Hispanic man with LGACC whose disease was managed with orbital exenteration and who is now being monitored for malignancy.

## Case presentation

A 32-year-old Hispanic man presented with a six-year history of a pressure sensation in the right orbit. He underwent a computed tomography (CT) scan for an unrelated problem, which incidentally revealed an enlarged right lacrimal gland. The patient was referred at that time to an ophthalmologist for further evaluation. However, he delayed seeking treatment due to various social circumstances. Approximately two years before the presentation, he began to develop pain, double vision, and lacrimation from the right eye (OD). Review of systems and social and family histories were otherwise unremarkable.

Upon a comprehensive ophthalmic evaluation, his best-corrected visual acuity was 20/20 in both eyes (OU). The intraocular pressure was 10 mmHg OD and 12 mmHg in the left eye (OS). The pupils were round and reactive to light, and there was no afferent pupillary defect. Ishihara color-plate testing was normal OU. Extraocular movements were within normal limits without restriction. The margin to reflex distance (MRD1) was 2.0 mm OD and 4.0 mm OS. The margin to reflex distance 2 (MRD2) was 5.0 mm OD and 5.0 mm OS. Hertel exophthalmometry values (base 93) were 18 OD and 16 OS. Hypoglobus of the right globe, with inferonasal displacement, was appreciated. External examination revealed a palpable mass and fullness in the right upper eyelid (**Figure 1**). Slit-lamp examination was within normal limits bilaterally. The patient’s fundus examination was unremarkable OU.

**Figure 1:**
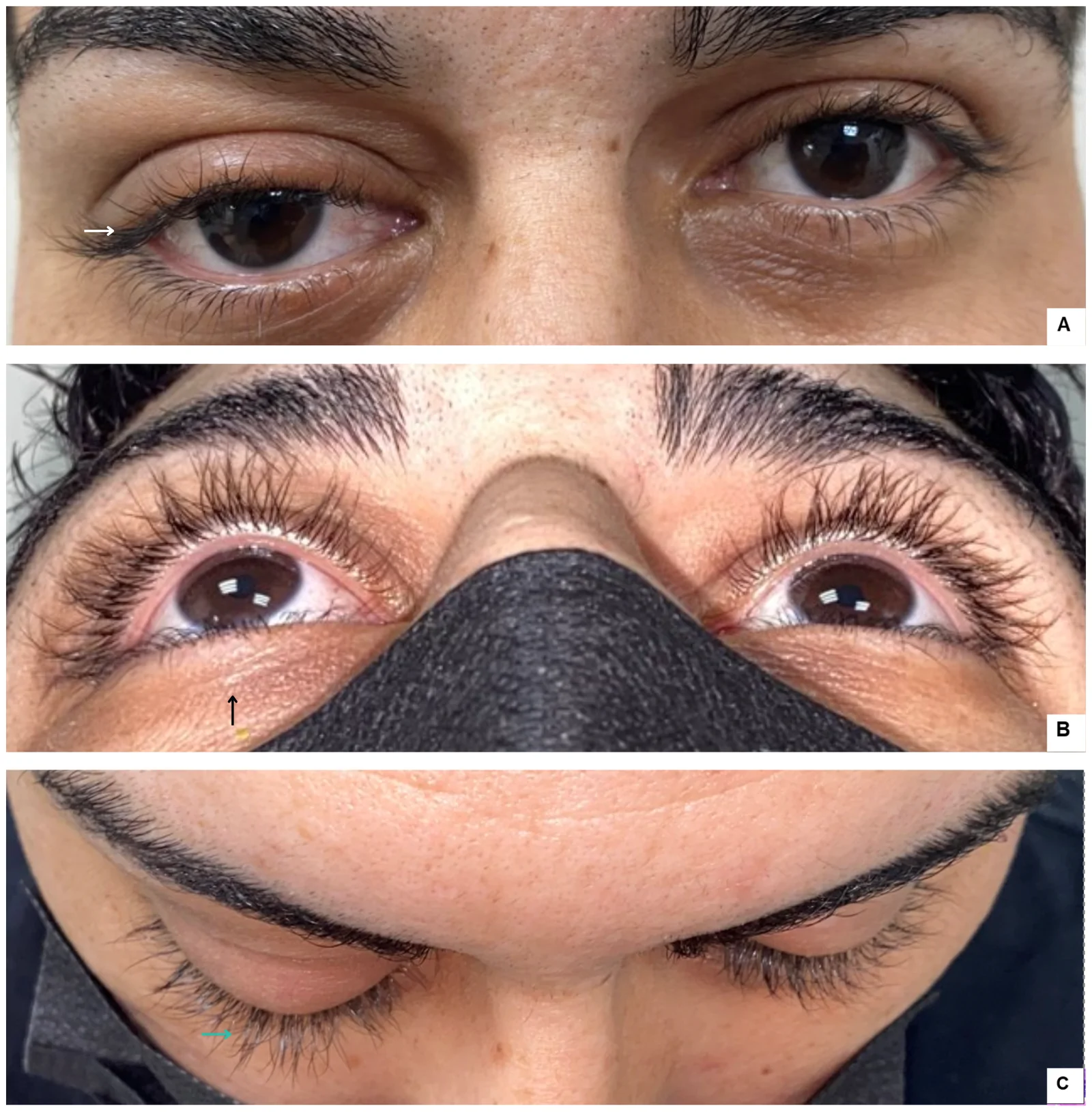
External photographs of the patient at presentation. **(A)** Anterior view showing inferonasal displacement (hypoglobus) of the right globe (white arrow) relative to the left globe. **(B)** Worm’s-eye view showing anterior displacement (proptosis) of the right globe (black arrow) relative to the left globe. **(C)** Bird’s-eye view showing anterior displacement of the right globe (green arrow) relative to the left globe.

An orbital computed tomography scan and facial magnetic resonance imaging (MRI) revealed an asymmetrical enlargement of the right lacrimal gland with a very well-defined heterogeneous enhancing solid mass measuring the following dimensions: 3.2 cm anteroposterior, 1.8 cm transversely, and 2.9 cm craniocaudal. Erosive osseous changes were appreciated in the lacrimal gland fossa, involving the roof and lateral wall of the right orbit. No intracranial extension of the mass was noted (**Figure 2**).

**Figure 2:**
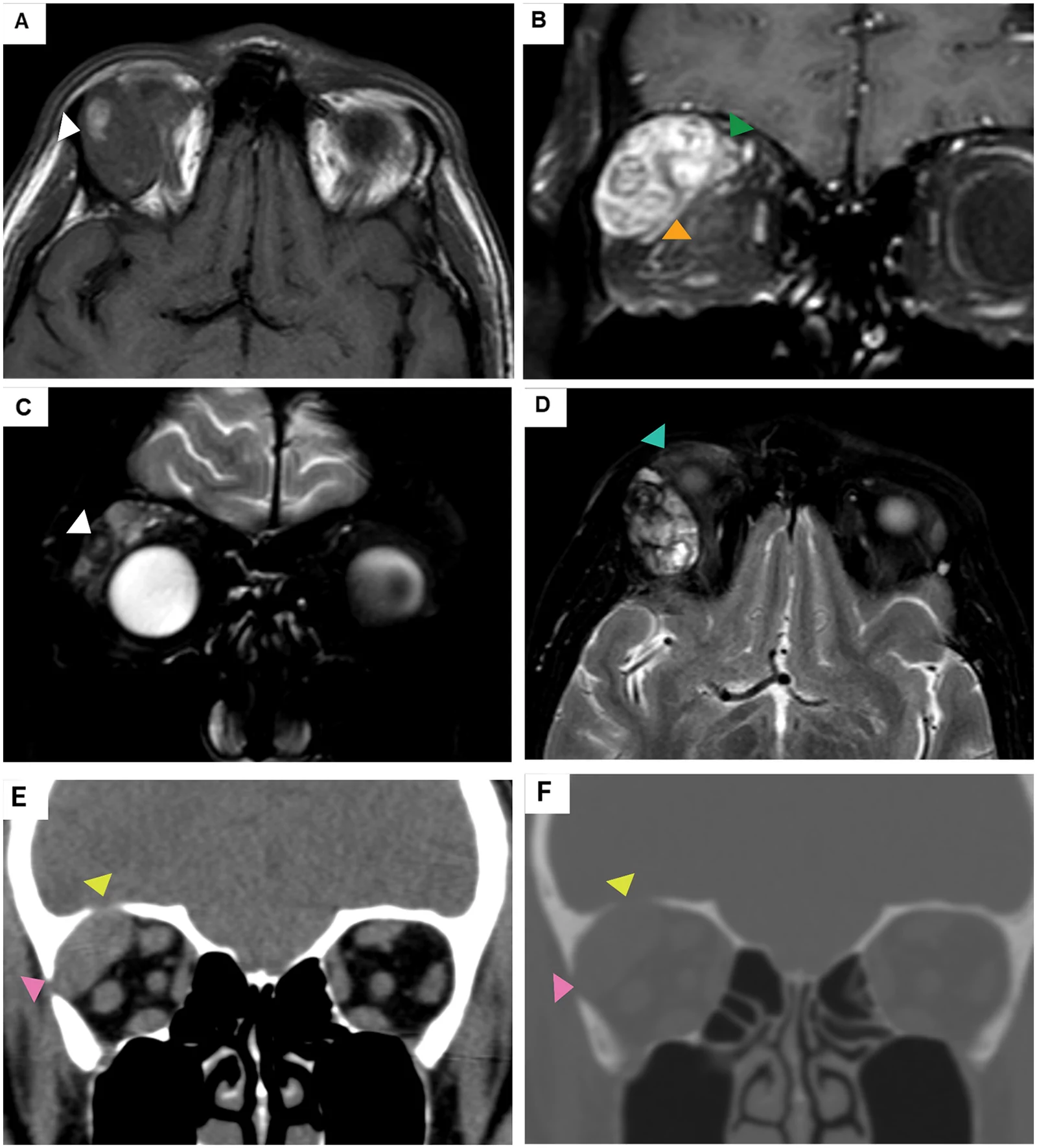
Radiological findings of the right lacrimal gland lesion. **(A)** Axial pre-contrast T1-weighted MRI showing a homogeneously hypointense lesion in the right lacrimal gland (white arrowhead) with internal foci of signal prolongation suggestive of hemorrhage. **(B)** Post-contrast coronal T1-weighted MRI showing avid heterogeneous enhancement of the right lacrimal gland lesion with mass effect on the medial rectus muscle (orange arrowhead) and superior rectus muscle (green arrowhead). **(C)** Coronal T2-weighted MRI showing a heterogeneously hypointense right lacrimal gland mass (white arrowhead) with markedly hypointense internal foci indicative of hemorrhagic products and internal cystic areas of T2 hyperintensity. **(D)** Axial T2-weighted MRI showing the right lacrimal gland lesion with associated proptosis of the right globe (blue arrowhead). **(E)** Coronal non-contrast CT of the orbits, soft-tissue window, showing a soft-tissue mass involving the right lacrimal gland with erosive/permeative osseous changes of the roof of the right orbit (yellow arrowhead) and of the lateral wall of the right orbit (pink arrowhead). **(F)** Coronal non-contrast CT of the orbits, bone window, at the same level, highlighting the erosive/permeative changes of the orbital roof (yellow arrowhead) and lateral orbital wall (pink arrowhead).

A biopsy was obtained from the anterior aspect of the right lacrimal gland for tissue diagnosis, with the lacrimal gland artery preserved. The diagnosis of adenoid cystic carcinoma was confirmed by immunohistochemistry, as the tumor cells were positive for CD117 and p63. The patient had a Ki-67 proliferation index of up to 70%, focally (**Figure 3**).

**Figure 3:**
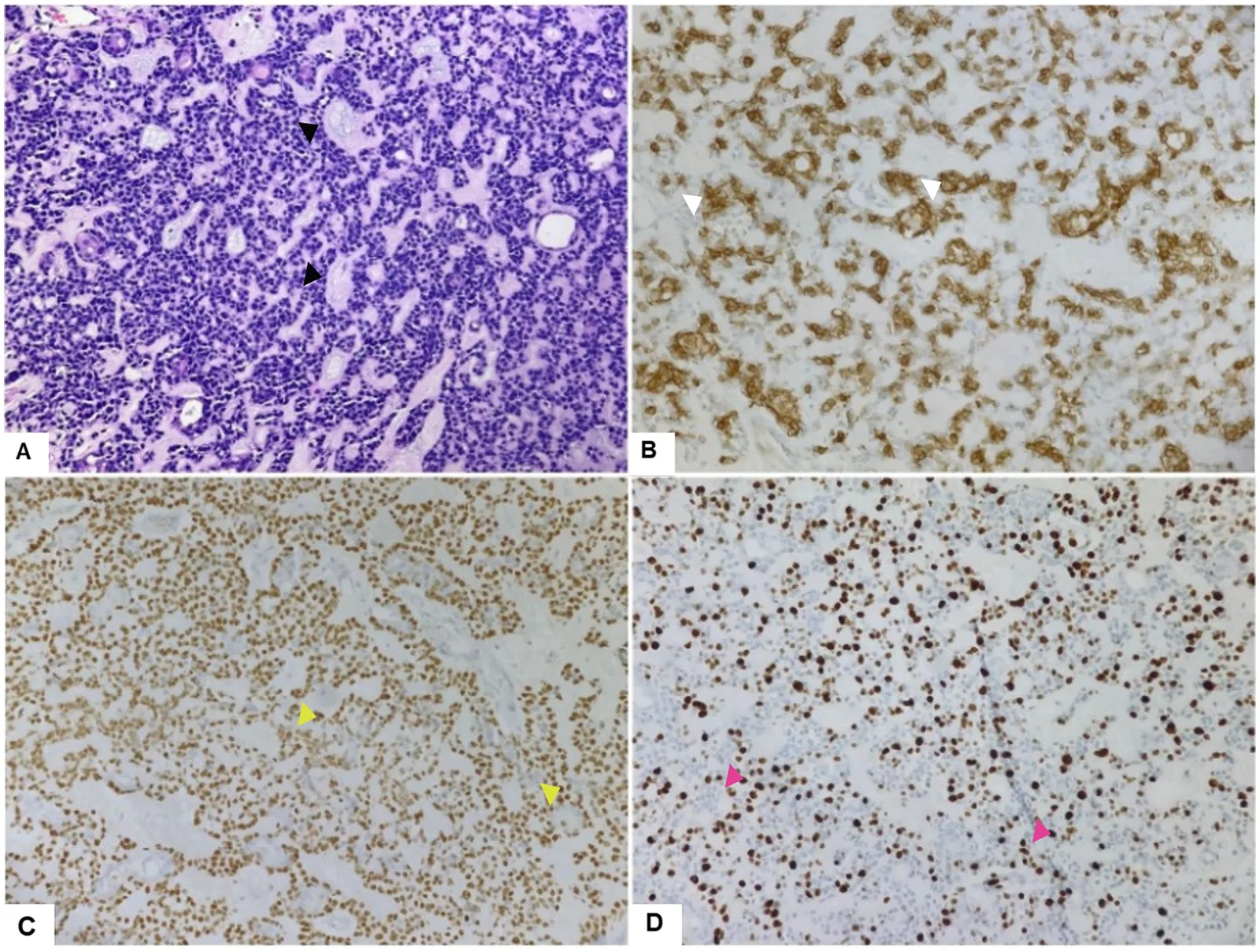
Incisional biopsy of the right lacrimal gland. **(A)** Variable-sized cystic spaces filled with basophilic material (black arrowheads); hematoxylin and eosin, original magnification 10×. **(B)** CD117 expression in tumor cells (white arrowheads); immunohistochemistry, 20×. **(C)** p63 nuclear expression in abluminal cells (yellow arrowheads); immunohistochemistry, 20×. **(D)** Ki-67 proliferation index positive in up to 70% of tumor cells (pink arrowheads); immunohistochemistry, 20×.

Baseline systemic evaluation for distant metastasis consisted of a chest radiograph, which showed no evidence of pulmonary metastasis. No CT of the chest or abdomen or PET-CT was performed. Regional lymph nodes were not formally assessed. A formal American Joint Committee on Cancer (AJCC) stage was not assigned at the time of treatment.

Subsequently, the patient was evaluated by the neurosurgery service, which found no evidence of intracranial spread. He was also evaluated by the hematology-oncology service for possible intra-arterial cytoreductive chemotherapy through the lacrimal artery. Intra-arterial cytoreductive chemotherapy was not available at our institution, and financial constraints precluded the patient from traveling out of state to receive this therapy. The multidisciplinary team therefore elected systemic neoadjuvant chemotherapy, administered intravenously, before definitive surgery. The patient received three cycles of doxorubicin 25 mg/m² intravenously plus cisplatin 25 mg/m² intravenously, each cycle consisting of three consecutive daily doses repeated every 21 days. Chemotherapy was complicated by myelosuppression that required hospitalization and was managed by the internal medicine service, with full recovery. Post-chemotherapy CT and MRI were performed, but no reduction in tumor size was found. Because of the extent of locally destructive disease, including erosion of the orbital roof and lateral orbital wall, a globe-sparing resection was considered unlikely to achieve oncologically sound margins, and orbital exenteration was recommended. Following multidisciplinary counseling regarding the morbidity of exenteration, the limitations of a globe-sparing approach, and the likely need for multimodal therapy regardless of the surgical choice, the patient elected to proceed with right orbital exenteration.

A pathological examination revealed a well-circumscribed tumoral mass in the lateral aspect of the orbit and involving the lacrimal gland. The tumor measured 4 x 3 x 1 cm. Grossly, the tumor did not involve any eye structures. On being sectioned, the tumoral mass was found to have a yellow-tan appearance and presented focal areas of hemorrhage and necrosis. Microscopically, the tumor demonstrated the presence of treatment effect with extensive necrosis, hemorrhage, fibrosis, and focal dystrophic calcifications, and scattered nodules of viable tumor comprising about 31%–40% of the entire mass, reflecting a partial pathological response to neoadjuvant chemotherapy. The specimen margins were negative for tumor cells, and there was no lymphovascular or perineural invasion identified (**Figure 4**).

**Figure 4:**
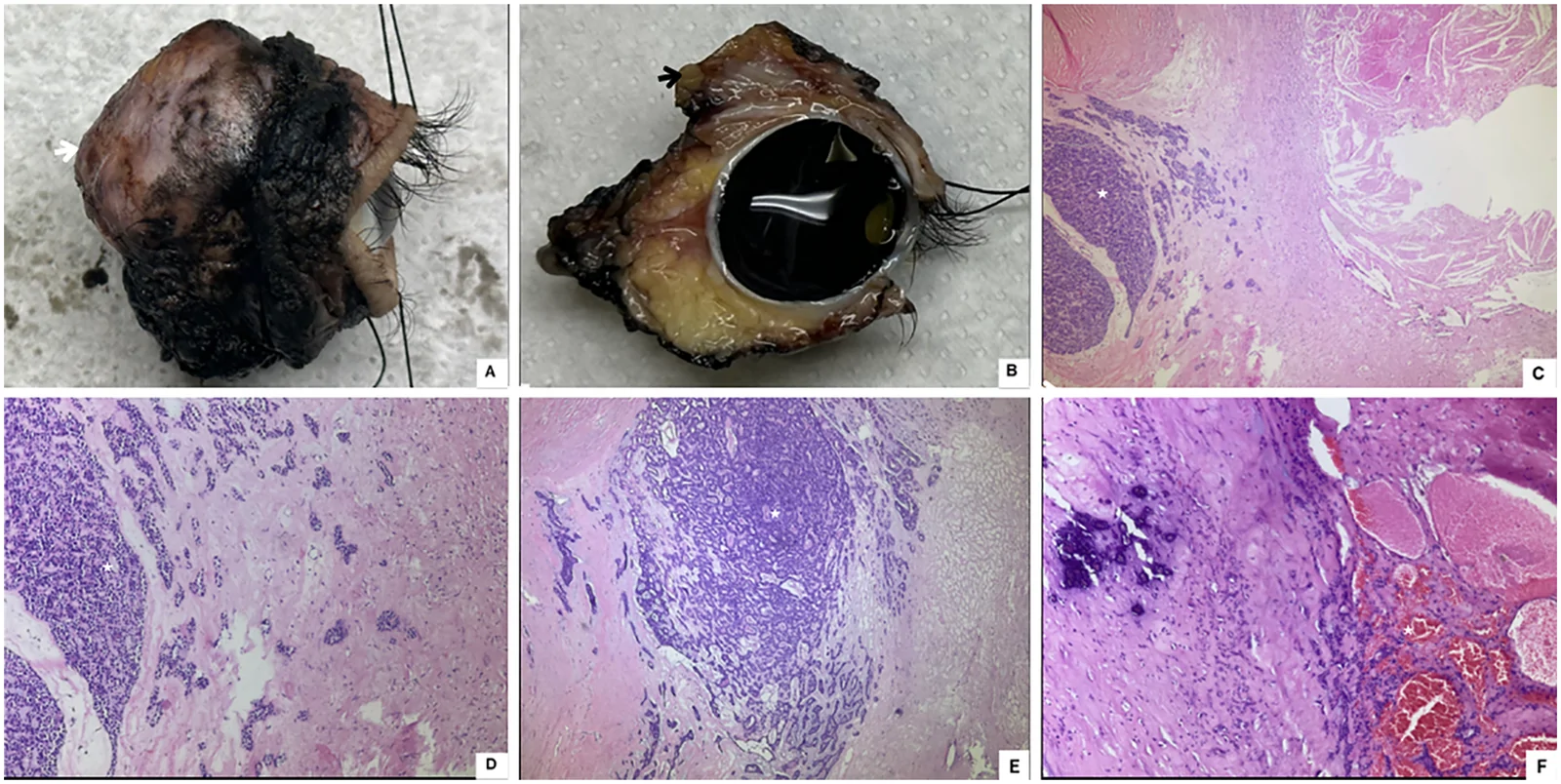
Right orbital exenteration specimen. Gross specimen showing a well-circumscribed mass (white arrow) in the region of the lacrimal gland. **(B)** Cut surface showing the mass (black arrow) with no involvement of the globe. **(C–F)** Microscopic images displaying residual tumor nests characterized by cribriform and tubular patterns, alongside marked post-therapy effects including fibrosis, hemorrhage, and focal dystrophic calcifications (area demarcated by a white star).

The patient then completed adjuvant radiotherapy to the right orbit and lacrimal gland tumor bed, receiving a total dose of 60 Gy in 30 fractions of 2 Gy over 42 elapsed days, delivered by volumetric modulated arc therapy (VMAT) with daily cone-beam CT guidance for image-guided radiotherapy (IGRT); treatment was completed 3.5 months after surgery. Radiotherapy was well tolerated; at completion, the only acute toxicity was residual erythema over the medial aspect of the orbit and inner nasal area.

An initial postoperative MRI of the orbit and brain, performed two months after exenteration and before completion of adjuvant radiotherapy, showed no definitive evidence of residual or recurrent tumor and no suspicious intracranial process. Subsequent surveillance orbital and brain MRIs at 18 and 25 months postoperatively showed no recurrent mass in the right orbital region and no intracranial abnormality. Systemic surveillance consisted of chest radiographs obtained at the same time points as the surveillance MRIs (2, 18, and 25 months postoperatively), none of which showed evidence of pulmonary metastasis. No formal quality-of-life instrument was administered during follow-up. At the most recent follow-up, 34 months after exenteration, the patient remained without evidence of local recurrence on orbital MRI or of pulmonary metastasis on chest radiography, had a well-healed socket, and was pursuing evaluation for a magnet-retained orbital prosthesis. The chronology of the episode of care is summarized in **Table 1**.

**Table 1:** Timeline of the episode of care.

Time point	Event
6 years before presentation	Onset of right orbital pressure sensation. Incidental finding of an enlarged right lacrimal gland on CT performed for an unrelated indication; referral to ophthalmology (evaluation deferred due to social circumstances).
2 years before presentation	Development of right orbital pain, diplopia, and lacrimation.
At presentation	Comprehensive ophthalmic evaluation: BCVA 20/20 OU; MRD1 2.0 mm OD; hypoglobus with inferonasal globe displacement; palpable right upper eyelid mass.
1 month after presentation	Orbital CT and facial MRI: well-defined, heterogeneously enhancing right lacrimal gland mass (3.2 x 1.8 x 2.9 cm) with erosive osseous changes of the lacrimal gland fossa involving the orbital roof and lateral orbital wall; no intracranial extension.
6 weeks after presentation	Incisional biopsy: adenoid cystic carcinoma (CD117+, p63+, Ki-67 up to 70% focally). Baseline systemic evaluation for distant metastasis: chest radiograph with no evidence of pulmonary metastasis; no chest/abdominal CT or PET-CT performed; regional lymph nodes not formally assessed; formal AJCC stage not assigned.
4 to 5 months after presentation	Neurosurgical evaluation: no intracranial spread. Hematology-oncology evaluation: intra-arterial cytoreductive chemotherapy not feasible.
5 to 6 months after presentation	Three cycles of doxorubicin 25 mg/m² IV plus cisplatin 25 mg/m² IV, three consecutive daily doses per cycle, repeated every 21 days. Complicated by myelosuppression requiring hospitalization; full recovery.
2 weeks after completing chemotherapy	Post-chemotherapy CT and MRI: no radiographic reduction in tumor size.
7 months after presentation	Right orbital exenteration. Pathology: 4 x 3 x 1 cm mass; 31%–40% viable tumor within the specimen (treatment effect); negative margins; no perineural or lymphovascular invasion.
2 months after exenteration	Initial postoperative MRI of the orbit and brain (obtained before completion of adjuvant radiotherapy): no definitive evidence of residual or recurrent tumor; no suspicious intracranial process. Chest radiograph: no radiographic evidence of pulmonary metastasis.
3.5 months after exenteration	Completion of adjuvant radiotherapy to the right orbit and tumor bed: 60 Gy in 30 fractions of 2 Gy over 42 days (VMAT with daily cone-beam CT guidance). Well tolerated; residual erythema of the medial orbit and inner nasal area at completion.
18 months after exenteration	Surveillance orbital and brain MRI: no recurrent mass in the right orbital region; no intracranial abnormality. Chest radiograph: no radiographic evidence of pulmonary metastasis.
25 months after exenteration	Surveillance orbital and brain MRI: no recurrent mass in the right orbital region; no intracranial abnormality. Chest radiograph: no radiographic evidence of pulmonary metastasis.
34 months after exenteration (most recent visit)	Ongoing multidisciplinary surveillance (ophthalmology, hematology-oncology, neurosurgery) with serial orbital and brain MRI and chest radiography. No evidence of local recurrence; no pulmonary metastasis on chest radiography. Well-healed exenteration socket. Evaluation for a magnet-retained orbital prosthesis in progress.

BCVA, best-corrected visual acuity; OU, both eyes; MRD1, margin to reflex distance; OD, right eye; AJCC, American Joint Committee on Cancer; IV, intravenous; VMAT, volumetric modulated arc therapy.

## Discussion

Lacrimal gland adenoid cystic carcinoma is a tumor that presents multiple challenges in clinical practice (2). Epidemiological data specific to the lacrimal gland are scarce. For context, adenoid cystic carcinoma as a whole accounts for approximately 1% of all malignancies involving the head and neck (3). Within the lacrimal gland it is the most common malignant epithelial tumor, and LGACC has been described mainly in adults in their fourth decade of life (3). The presenting symptoms may include pain, diplopia, swelling of the lacrimal gland, hypoglobus, exophthalmos, or ptosis (3). The patient discussed in this report presented with a six-year history of pressure sensation and fullness of the orbit and subsequently developed pain, double vision, and lacrimation in the right eye approximately four years later. Other reports in the literature have estimated that the mean of the duration of symptoms before ophthalmic consultation was six months (3). Notably, the onset of our patient’s symptoms preceded his first ophthalmic evaluation by six years, an unusually long interval for a malignancy in which reported 5-year overall survival ranges from approximately 50% with surgery alone to 67%–78% with multimodal treatment (9). Even though LGACC tumors are characterized by extremely aggressive and invasive behavior, the patient in this case presented with locoregionally confined disease: a lacrimal gland mass with erosive involvement of the orbital roof and lateral orbital wall, but without intracranial extension and without radiographic evidence of distant metastasis. This characterization must be qualified by the limitations of the systemic evaluation, which consisted of chest radiography only, without chest or abdominal CT or PET-CT, and without formal assessment of the regional lymph nodes.

In the diagnostic assessment of this patient, the differential diagnosis of a lacrimal gland fossa mass included pleomorphic adenoma, other malignant epithelial tumors, lymphoproliferative disease, and inflammatory dacryoadenitis, including IgG4-related disease and sarcoidosis (5, 6). The pain, the erosive osseous changes on CT, and the heterogeneous enhancement on MRI raised concern for a malignant rather than a benign or inflammatory process, as pain and bone destruction, although not pathognomonic, are more commonly associated with adenoid cystic carcinoma, whereas pleomorphic adenoma tends to remodel rather than erode bone (3, 5, 7). Because clinical and imaging features alone cannot reliably distinguish these entities, an incisional biopsy was required to establish the diagnosis.

Despite the concerning clinical and imaging findings, the exenteration specimen showed several favorable histopathological features. The tumor exhibited predominantly cribriform and tubular, rather than solid, architecture—features associated with a more favorable prognosis in LGACC (5, 8). In addition, despite approximately six years of symptoms, the disease remained locoregionally confined, without intracranial extension or radiographic evidence of distant spread, and the exenteration specimen showed negative margins with no perineural or lymphovascular invasion. However, the prolonged symptom duration should be interpreted cautiously. Delayed presentation, influenced by social circumstances despite a previously documented incidental imaging finding, may partly explain the apparent long disease course. Furthermore, a focally elevated Ki-67 index of up to 70% argues against uniformly low proliferative activity. Therefore, this single case cannot establish an indolent tumor variant; stage at presentation and histologic subtype remain more important prognostic factors than symptom duration alone in LGACC (5, 9). The discrepancy between the high proliferative activity observed in the biopsy and the more favorable features of the final pathology may reflect sampling of a focal high-proliferation area, a preferential cytotoxic effect of chemotherapy, or both.

This patient underwent three cycles of systemic therapy with cisplatin and doxorubicin, followed by exenteration of his right orbit. Tse et al. report improvement in overall survival with intra-arterial cytoreductive chemotherapy before exenteration, a benefit recently corroborated in a population-based comparison (10, 11). When intra-arterial delivery is unavailable, as in our patient, the choice lies between proceeding directly to surgery and attempting systemic neoadjuvant cytoreduction. We favored the latter in order to reduce tumor burden, facilitate complete resection with negative margins, and treat occult regional disease. Intravenous cisplatin and doxorubicin preserve the rationale of the intra-arterial regimen even though the high local drug concentration achieved by intra-arterial infusion cannot be reproduced (10, 13), and platinum/anthracycline-based combinations remain the most widely used cytotoxic regimens for adenoid cystic carcinoma (12).

There is currently a lack of certainty regarding the optimal treatment for this disease (1–4). Various case reports present different strategies, including exenteration, eye-sparing surgery, chemotherapy, and radiation therapy (2–4). Some authors suggest that the survival rate in patients who are treated with exenteration is similar to that in those who undergo eye-sparing surgery (2, 3, 9). In our patient, the bone erosion of the orbital roof and lateral wall was decisive. Preservation of the globe would have entailed a substantial risk of residual microscopic disease within the orbit and adjacent bone, with subsequent local progression and potential extension toward the intracranial compartment. The therapeutic goal therefore shifted from organ preservation to maximal locoregional control. This decision was also consistent with the surgical component of the trimodal protocol of Tse et al. (neoadjuvant intra-arterial cytoreductive chemotherapy, orbital exenteration, and adjuvant chemoradiotherapy), which has among the most mature long-term outcome data for LGACC (10, 13). Nevertheless, LGACCs tend to recur in the original location or as distant metastasis (4).

The lack of radiographic response in this case is consistent with the known chemoresistance of adenoid cystic carcinoma, in which objective response rates to platinum- and anthracycline-based systemic regimens are low and typically short-lived (5, 8, 12). Notably, despite the absence of radiographic shrinkage, the exenteration specimen showed extensive necrosis and fibrosis with only 31%–40% viable tumor, indicating a radiographically occult cytotoxic effect. This proportion of viable tumor within the resected specimen is a measure of pathological response. In addition, this patient had negative surgical margins and no perineural invasion—both favorable features given the strong association between margin status, perineural invasion, and recurrence risk in LGACC (5, 9, 13, 14). Neoadjuvant chemotherapy thus offers potential cytoreduction at the cost of toxicity and delayed definitive surgery.

Adjuvant radiotherapy is now a near-constant component of multimodal management of LGACC, whether or not the globe is preserved. In eye-sparing series, postoperative radiotherapy has been associated with significantly better recurrence-free survival (15, 16), although outcomes remain poor in T3–T4 disease because of distant failure (17). Radiotherapy has also been reported at the case level, including postoperative VMAT after eye-sparing surgery (18) and after orbital exenteration (3). Proton therapy has achieved long-term globe preservation in selected patients (19, 20) but is not widely available. In our patient, the erosive bone involvement and the focally high Ki-67 index argued for adjuvant irradiation despite negative margins and the absence of perineural invasion. The 60 Gy dose is consistent with the postoperative literature (16), and because the globe had been removed, the ocular toxicities of eye-sparing protocols did not apply.

Patients with LGACC may require lifelong close monitoring. There is a lack of certainty regarding the specific studies that need to be performed and the optimal timing for follow-up. Nevertheless, physicians should tailor management strategies to each patient’s individual findings. Further studies should be conducted to develop improved algorithms for monitoring morbidity and mortality in patients with LGACC.

This report has limitations: as a single case with a follow-up of 34 months, short relative to the decade-long recurrence horizon of adenoid cystic carcinoma, it cannot support conclusions about treatment efficacy. Systemic surveillance during follow-up was limited to chest radiography, which is less sensitive than CT for small pulmonary metastases. Consequently, the absence of pulmonary metastasis reported here should be understood as the absence of radiographically detectable disease on chest radiography, and occult micrometastatic disease cannot be excluded; cross-sectional imaging of the chest is now incorporated into the patient’s ongoing surveillance. Molecular characterization, such as MYB-NFIB fusion testing, was not performed; and no validated quality-of-life instrument was administered, so the functional and psychosocial impact of exenteration could not be quantified.

## Patient perspective

The patient expressed concern about eye removal but felt reassured by clear communication and regular updates throughout treatment.

## Conclusions

Lacrimal gland adenoid cystic carcinoma is a rare, aggressive, and life-threatening malignancy. The treatment of LGACC requires an individualized, interdisciplinary, and multimodal approach. Diverse strategies for treating LGACC exist, but there is no consensus about which is the best; what can be agreed upon, however, is that long-term patient monitoring is critical and future research efforts should aim to establish more precise guidelines for surveillance and management to enhance patient outcomes.

## Data Availability

The original contributions presented in the study are included in the article/supplementary material. Further inquiries can be directed to the corresponding author.
